# Alterations in the Secretome of Clinically Relevant Preparations of Adipose-Derived Mesenchymal Stem Cells Cocultured with Hyaluronan

**DOI:** 10.1155/2015/421253

**Published:** 2015-07-15

**Authors:** Peter Succar, Edmond J. Breen, Donald Kuah, Benjamin R. Herbert

**Affiliations:** ^1^Department of Chemistry and Biomolecular Sciences, Macquarie University, Office 256, Building E8C, Balaclava Road, North Ryde, NSW 2109, Australia; ^2^Royal North Shore Hospital, University of Sydney, St Leonards, NSW 2065, Australia; ^3^Australian Proteome Analysis Facility, Macquarie University, North Ryde, NSW 2109, Australia; ^4^Sydney Sports Medicine Centre, 6 Figtree Drive, Sydney Olympic Park, NSW 2127, Australia; ^5^Regeneus Ltd., 25 Bridge Street, Pymble, NSW 2073, Australia

## Abstract

Osteoarthritis (OA) can be a debilitating degenerative disease and is the most common form of arthritic disease. There is a general consensus that current nonsurgical therapies are insufficient for younger OA sufferers who are not candidates for knee arthroplasties. Adipose-derived mesenchymal stem cells (MSCs) therapy for the treatment of OA can slow disease progression and lead to neocartilage formation. The mechanism of action is secretion driven. Current clinical preparations from adipose tissue for the treatment of OA include autologous stromal vascular fraction (SVF), SVF plus mature adipocytes, and culture-purified MSCs. Herein we have combined these human adipose-derived preparations with Hyaluronan (Hylan G-F 20: Synvisc) *in vitro* and measured alterations in cytokine profile. SVF plus mature adipocytes showed the greatest decreased in the proinflammatory cytokines IL-1*β*, IFN-*γ*, and VEGF. MCP-1 and MIP-1*α* decreased substantially in the SVF preparations but not the purified MSCs. The purified MSC preparation was the only one to show increase in MIF. Overall the SVF plus mature adipocytes preparation may be most suited of all the preparations for combination with HA for the treatment of OA, based on the alterations of heavily implicated cytokines in OA disease progression. This will require further validation using *in vivo* models.

## 1. Introduction

Osteoarthritis (OA) can be a debilitating degenerative disease and is the most common form of arthritic disease. Onset is usually in the third and fourth decade of life and has a gradual worsening prognosis over time.

OA is most prevalent in weight bearing joints; the highest incidence of the disease occurs in the knee. It is classified as idiopathic and or secondary. Primary clinical intervention currently involves limited systemic pharmacotherapy such as analgesics and nonsteroidal anti-inflammatory drugs (NSAIDs). Controlling mechanical overload through weight loss and supporting braces can be used autonomously or in combination with physical therapy. Viscosupplementation can also be used to treat OA. This aims to replace lost synovial fluid in an OA knee with Hyaluronan (HA) to reduce pain and increase mobility by lubricating the joint. HA is an endogenous polysaccharide found in all tissues and body fluids of vertebrates. HA is especially abundant in loose connective tissue and is a major component of cartilage [1] and the synovium [2]. Aside from its rheological properties, high molecular weight HA can decrease apoptosis, oxidative stress, and necrosis [3].

Arthroscopic surgical interventions are used for the debridement of mechanical cartilage tears, lesions, and defects, as well as cartilage resurfacing or microfracture to stimulate fibrocartilaginous repair. However, due to inherent degenerative factors of OA and the limited capacity of conventional therapies to halt disease progression, arthroplasties (artificial joint replacement surgery) are commonly performed for a large proportion of OA patients. The useful lifetime of an artificial knee joint is between 10 and 20 years. Average time for revision surgery has been reported at 35 months and is most commonly caused by infection, instability, and stiffness [4]. For these reasons arthroplasties are often not recommended for patients under 60. There is a general consensus that current nonsurgical therapies are insufficient for younger OA sufferers who are not candidates for knee arthroplasties [5]. Although current therapies may provide temporary pain relief and increased mobility, there is a pressing need for treatments that slow or stop the degradation of cartilage.

The use of alternative biological therapies, such as mesenchymal stem cells (MSCs), for the treatment of OA is increasing as a result of a gap in healthcare options for middle age patients [5, 27]. MSCs can differentiate into mesodermal lineages making them an attractive source for cell based therapies in musculoskeletal conditions such as OA [28]. However, differentiation is not the primary mode of action and* in vitro* animal models and human data have demonstrated that a complex set of secretions drive the anti-inflammatory and regenerative effects that have been observed [29]. Thus, the secretome of MSCs has become of particular interest and multiplex cytokine analyses are used to assess cell populations and the “cytokine profile” is predictive of* in vivo* effect [30]. Proinflammatory cytokines such as Interleukin-1*β* (IL-1*β*) play a pivotal role in the pathogenesis of OA. IL-1*β* can dramatically increase the expression of matrix metalloproteinases, which contribute to cartilage degradation in OA. Chondrocytes stimulated with IL-1*β* increase secretion of Nitric oxide and Prostaglandin E2, which are key mediators essential to inflammation in OA [31].

The role of inflammation in the pathophysiology of OA has shifted from originally being considered a by-product of the disease to the current understanding as a key driver of disease progression [32]. MSCs have an anti-inflammatory effect and are able to stimulate cartilage proliferation via paracrine signaling [33]. As such, regenerative therapies using MSCs are emerging as a suitable treatment for degenerative musculoskeletal defects.

Surgeons have experimented with possible combination therapies; most notably Lee et al. showed in a porcine model using histopathological observation that injection of bone marrow-derived MSCs suspended in Hyaluronan (Hylan G-F 20: Synvisc) could home to the site of injury, adhere, proliferate, and lead to neocartilage formation [34]. One limitation of this study as concluded by the authors was that regeneration may have been limited by a dose-dependent response to the number of MSCs injected.

One of the first MSCs sources to be isolated came from bone marrow, however as shown by Kern et al. in a comparative analysis between adipose and bone marrow-derived, these MSCs may be inferior to adipose-derived MSCs, in terms of clinical feasibility. It was shown that adipose tissue had the highest concentration of MSCs and a greater proliferative capacity when compared to bone marrow-derived MSCs [35]. The above components combined with minimal invasiveness spearhead adipose tissue as a favorable source of MSCs in emerging regenerative therapies.

Hyaluronan (HA) shows great potential as an adjunct to adipose-derived MSC regenerative therapy for the treatment of OA not only for its ability to provide rheological cushioning but also for the mechanistic biochemical relationship it forms with MSCs. The relationship with high molecular weight HA and MSCs is founded in CD44, the primary HA receptor, and abundantly expressed on the surface of MSCs. It has been proposed that the cross-talk between CD44 and HA may promote rapid resolution of immune responses [36] and thus enhance the anti-inflammatory effect of MSCs in OA.

In this study we sought to assess the feasibility of a range of preparations in which adipose-derived mesenchymal stem cell therapy could be administered in combination with HA by assessing the secreted soluble protein fraction produced by MSCs that is the secretome. We employed multiplex analyses of 48 cytokines involved in OA to assess the suitability of conditions. These conditions include autologous therapies such as the stromal vascular fraction (SVF), the SVF coinjected with autologous mature adipocytes, and cultured allogeneic preparations such as culture purified adipose-derived MSCs. As adipose-derived MSC therapy moves closer to mainstream medicine, secretome data will be a valuable assessment tool to inform animal studies and human clinical trials in years to come.

## 2. Materials and Methods

### 2.1. Isolation of Human Adipose-Derived Mesenchymal Stem Cell Populations

This research was approved by the Macquarie University human research ethics committee (Reference number 5201100385). Human lipoaspirate was obtained from patients undergoing elective cosmetic liposuction surgery. The lipoaspirate was digested as previously described [37]. Briefly, 200 mL of fresh lipoaspirate was mixed and enzymatically digested in prewarmed (37°C) saline containing 0.5 mg/mL collagenase (Lomb Scientific, USA). The lipoaspirate was then incubated in a 37°C water bath for 30 minutes and mixed periodically to circumvent layer separation. The digested lipoaspirate was then passed through an 800 *μ*m mesh to exclude undigested tissue clumps. Finally, the suspension was centrifuged at 1500 ×g for 5 minutes to obtain the pelleted stromal vascular fraction cells (SVF) and floating adipocytes. At this point, the SVF was either used for experimentation or propagated to obtain an adherent purified adipose-derived mesenchymal stem cell (MSC) population.

### 2.2. Propagation of Adherent Adipose-Derived Mesenchymal Stem Cells

To obtain a population of adherent MSCs, the SVF pellet was transferred into three T175 cm^2^ flasks containing standard culture media which consisted of Dulbecco's Modified Eagle Medium (DMEM; Invitrogen, USA) supplemented with 10% foetal bovine serum (FBS; Bovogen, Australia) and 1% penicillin-Streptomycin solution (Invitrogen, USA). Flasks were incubated for 72 hours at 37°C with 5% carbon dioxide. To prevent iron toxicity to adherent MSCs, the flasks were washed using DMEM with no additives to wash away nonadherent cells and the media replaced with fresh standard culture media. Once the monolayer of MSCs reached 80% confluency, cells were passaged with enzymatic digestion using TrypLE express (Invitrogen, USA) and then resuspended in standard culture media. Cells from each flask were then transferred to three new T175 cm^2^ flasks now termed passage 1. Media changes were performed every 72 hours.

Standardized enumeration of the SVF, dependent on nucleated cell clusters, was achieved with Trucount tubes (Becton Dickinson, USA) containing sheath fluid (isoflow; Becman Coulter, USA) and nucleic acid dyes; propidium iodide (10 *μ*g/mL; Sigma, USA), Syto11 (1 *μ*M; Invitrogen, USA), and a defined bead number. The combination of a charged (propidium iodide (Pi)) and a cell permeate (Syto11) dye allows for discriminant cell population gating, which are based on the principal of Pi membrane exclusion and thus viability can be calculated from defined bead populations run as standards. Samples were all run on FACSCalibur flow cytometer (Becton Dickinson, USA).

### 2.3. SVF Seeded with Hyaluronan Treatment

Following on from the digestion of the fresh lipoaspirate, the pelleted SVF was resuspended in standard culture media. Viable SVF cells were all seeded at two million nucleated cells per T175 cm^2^ flask with or without the addition of adipocytes. Flasks were always seeded in either two conditions: the control, which consisted of standard culture media, or treatment, which consisted of HA media (standard culture media; hyaluronan (6% (v/v))). In each condition, three subset arms were tested; SVF and mature adipocytes, SVF alone, and mature adipocytes alone. Adipocytes were used at 2.5 mL (suspended in saline) and flasks were normalized to 20 mL total containing 17.5 mL media and 2.5 mL saline equivalent (with or without adipocytes). Flasks were incubated for 72 hours at 37°C with 5% carbon dioxide.

### 2.4. Purified Adherent-MSC Hyaluronan Treatment

#### 2.4.1. Seeding Experiment

Once the flasks reached 80% confluency at passage 2, cells were stripped off the plastic and enumerated. Viable MSCs were then seeded at 1.1 million cells per T175 cm^2^ flask. Total media contained within the flasks was standardized to 20 mL. Flasks were seeded with standard culture media or HA media for treatment flasks. This method approximates the intra-articular coinjection of HA and MSCs. After 24 hours of incubation, conditioned media was collected from all flasks. Cells were then stripped off the plastic and a portion was used for immunophenotypic characterization.

#### 2.4.2. Media Change Experiment

Once the flasks reached 80% confluency at passage 2, cells were stripped off the plastic and enumerated. Cells were seeded at 1.1 million cells per T175 cm^2^ and incubated in standard culture conditions. On the third day the media was changed with either standard culture media in control flasks or HA media for treatment flasks. After three days, conditioned media was collected from each of the flasks. Cells were then stripped off the plastic and a portion was used for immunophenotypic characterization.

### 2.5. Morphology and Differentiation

#### 2.5.1. Morphology

Prior to harvesting the purified adherent-MSCs were imaged under a Zeiss Primo Vert inverted light microscope (Zeiss, Australia) using an eye-piece attached, Canon EOS 5D Mark II Digital SLR camera (Canon, Australia).

#### 2.5.2. Differentiation

Adipogenic and osteogenic differentiation media formulations were used as previously described [38] with slight modification. In brief, cells received media changes twice weekly for 28 days. Upon termination of the differentiations, monolayers were washed in PBS and fixed in 4% paraformaldehyde for 1 hour. To confirm the multipotency of our MSCs, differentiated adipocytes and osteocytes were challenged with Oil Red O (Sigma, Australia) and Alizarin Red (Sigma, Australia), respectively.

The adipogenic differentiation was washed with milliQ water, incubated with 60% isopropanol, stained using three parts filtered Oil Red O stock (0.3% w/v) with two parts milliQ water for five minutes at room temperature, and washed with tap water. The osteogenic differentiation was washed with milliQ, stained with 2% Alizarin Red S solution for two minutes at room temperature and washed three times with milliQ water.

All differentiations were visualized using an Olympus IMT-2 inverted microscope (Olympus, Australia) and imaged with a mounted Scion VisiCapture Firewire camera (Scion Corporation, USA).

### 2.6. Immunophenotypic Characterization

MSC characterization can be achieved through adherence to cell culture flasks, differentiation, and immunophenotype. A portion of the endpoint cells collected was used for MSC immunophenotypic characterization. Cells from either control or treated flasks were diluted in their respective media (standard culture media or HA media) and centrifuged at 2000 ×g for 5 minutes. The cells were washed in PBS and resuspended in PBS with 2% FBS. The cells were stained with the following antibodies, which were all sourced from BD Biosciences: CD34 (number 550619), CD45 (number 557059), CD73 (number 550257), CD90 (number 555596), CD105 (number 560839), and IgG1_K_ isotype control (number 551436). Cell were incubated with each of the antibodies for 45 minutes and then washed with ice cold PBS, centrifuged at 2000 ×g for 5 minutes and resuspended in 1x FACS Lysing Solution (Becton Dickinson, USA). The cells were all stained with phycoerythrin (PE) conjugated antibodies and thus were resuspended in Syto11 (1 *μ*M) and isoflow to achieve contrast fluorescence. Stained and unstained control cells were analyzed using a FACSCalibur flow cytometer (Becton Dickinson, USA). CD44 has long been recognized as an established receptor for HA [39]; therefore in addition to the immunophenotypic MSC characterization above, CD44 (number 550989) was also tested using the same protocol.

### 2.7. Secretome Analysis

The conditioned media was collected from every flask in this study, centrifuged at 5000 ×g for 5 minutes, and stored at −80°C. Upon thawing, the samples were filtered through 0.2 mm Nanosep MF Centrifugal Devices with Bio-InertH Membrane (Pall Scientific, USA). Filtrates (50 *μ*L) were analyzed using either the Bio-Plex Pro Human Cytokine 27-plex or the Bio-Plex Pro Human Cytokine 21-plex assay (Bio-Rad, USA), according to the manufacturer's instructions. The washing steps were performed using the Bio-Plex Pro II magnetic wash station and the data was acquired using the Bio-Plex 200 system with version 5.0 software (Bio-Rad, USA). The average concentration of each cytokine in the conditioned medium samples was calculated from four technical replicates in cultured MSC experiments. To account for biological variation in the concentration of secreted cytokines and, in the stromal vascular fraction, the variable concentration of MSCs, the data was normalized to fold change of concentration (pg/mL) of HA treated over control (*n* = 3). The fold change was then averaged and graphed with the upper and lower confidence intervals set at 95% as error bars. Cytokines with a fold change less than one indicate a decrease in concentration with HA media treatment, a fold change greater than one indicates an increase in the cytokine with HA treatment. Any cytokines with a fold change clear of the axis at 1 were reported as significant (determined numerically).

### 2.8. Experimental Schematic

See Figure 1.

## 3. Results

### 3.1. Multipotent MSC Validation

Previously published data from our lab demonstrated methods used within this study to both isolate and culture/propagate the SVF and obtain adherent-MSC populations [37, 38]. These were in accordance with the Mesenchymal and Tissue Stem Cell Committee of the International Society for Cellular Therapy statement [40]. Herein we have supplemented media used to grow MSCs with HA media in an attempt to quantify the effects on the secretome.

**Figure 2:**
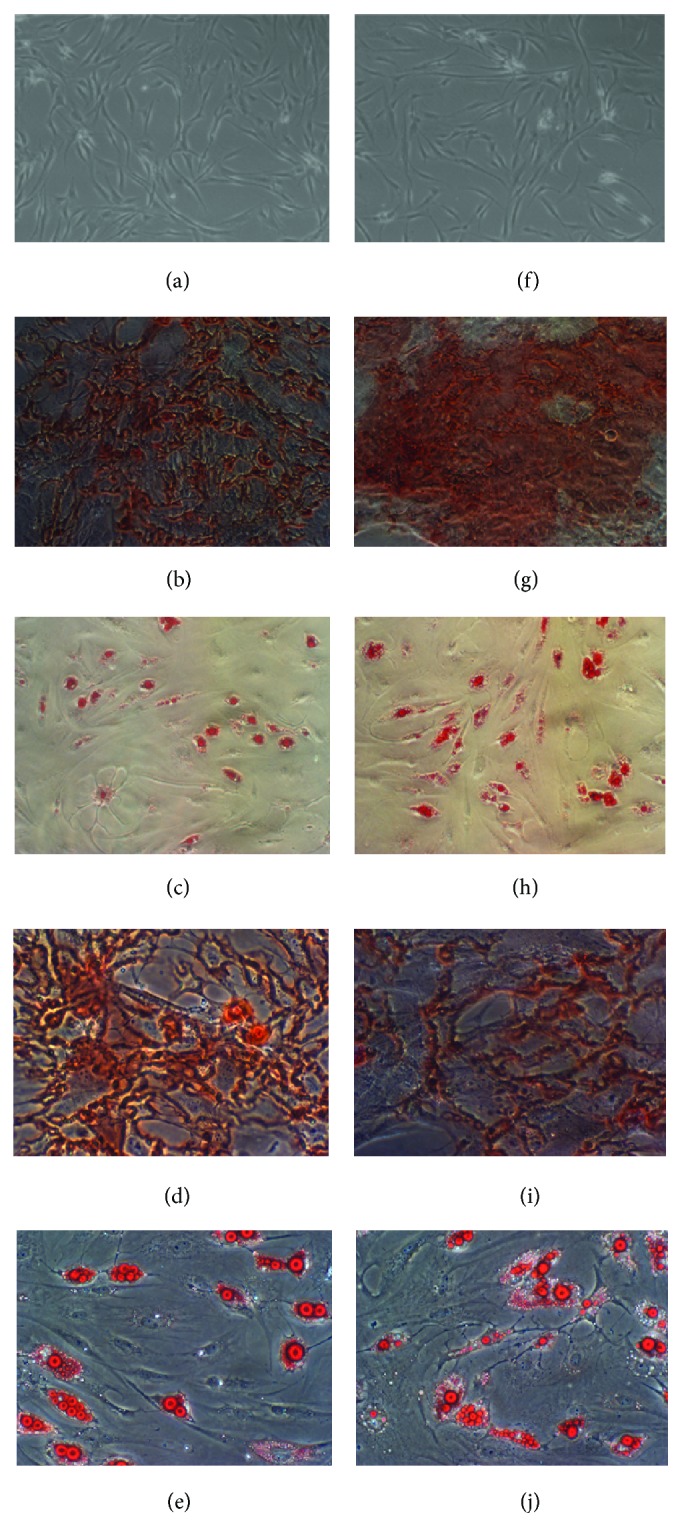
Morphology and differentiation potential of control and treated MSCs. MSCs cultured in either standard culture media or HA media. No detectable differences were seen in the morphology of control (a) MSCs and HA treated (f) monolayers. Osteogenic differentiation of control MSCs (b) and of the HA treated (g) at 10x magnification. Adipogenic differentiation of control (c) and of the HA treated (h) at 10x magnification. At 20x magnification no detectable differences in Alizarin Red S staining were observed in the osteogenic differentiation of control MSCs (d) and HA treated (i). At 20x magnification no detectable differences in Oil Red O staining were observed in the adipogenic differentiation of control MSCs (e) and HA treated (j).

Cultured MSCs were tested for immunophenotypic expression with and without HA media treatment. The MSC profile was maintained for the duration of the study. Monolayers from both the control and HA treated MSCs presented with equivalent differentiation potential.  Figure 2 shows no detectable difference in morphology and differentiation potential between MSCs cultured in standard culture media and HA media. The differentiated MSCs were tested with either Alizarin Red S, which stains positive for calcium deposits confirming osteogenic potential, or Oil Red O, which stains positive for lipid accumulation confirming adipogenic potential. The consistency in the take-up of both stains used to test differentiated cell monolayers indicates the differentiation potential is maintained.

**Figure 3:**
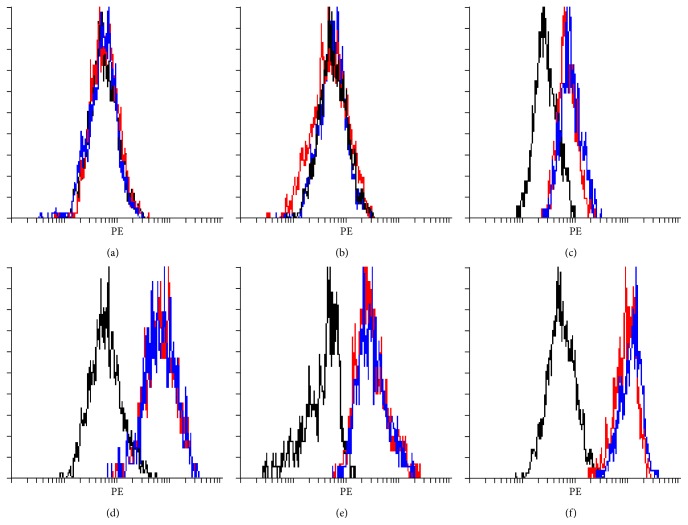
Immunophenotypic expression of control and HA treated MSCs. Immunophenotypic expression of MSCs cultured in either standard culture media or HA media. Each of the histograms depicts the IgG iso-type control (black dashed lines), control media (red dashed lines), and HA media (blue dashed lines). Maintenance of MSC profile was confirmed with the lack expression in the haematopoietic markers CD34 (a), CD45 (b), and positive expression of CD73 (c), CD90 (d), and CD105 (e) in both the control and HA treated MSCs. CD44 (f), the major receptor for HA, showed no major increases in immunophenotypic expression.

In addition to differentiation potential, immunophenotypic expression on the control and HA treated MSCs is depicted in Figure 3. The presence of CD73, CD90, and CD105 and the absence of hematopoietic markers CD34 and CD45 indicated the presence of replicative and self-renewal capacity of MSCs of both the control and the HA media treated. CD44 is a major receptor for HA; as such it was also tested. The number of CD44^+^ cells in the control and the HA media treatment showed no major quantifiable difference in the tested replicates.

### 3.2. Alterations of Secretion Profile in HA Media Treatment

In this study we sought to profile changes in the secretome to ascertain the effects of combining HA with MSCs. Changes in the secretions profile can be an indication of positive or negative effect in the context of OA; that is, increase in the anti-inflammatory cytokines may be considered positive or an increase in proinflammatory cytokines may be negative. All the cytokines were represented as a percentage of the control, that is, fold change (fold Δ). The average fold change of three biological replicates for each of the cytokines was plotted as an average ± the upper and lower confidence intervals at 95%. The horizontal axis was set at one (i.e., no change), and any point with error bars that cleared one was considered a significant difference from the control (determined numerically; see Table 1 for numerical summary of cytokine data).

### 3.3. Stromal Vascular Fraction

**Figure 4:**
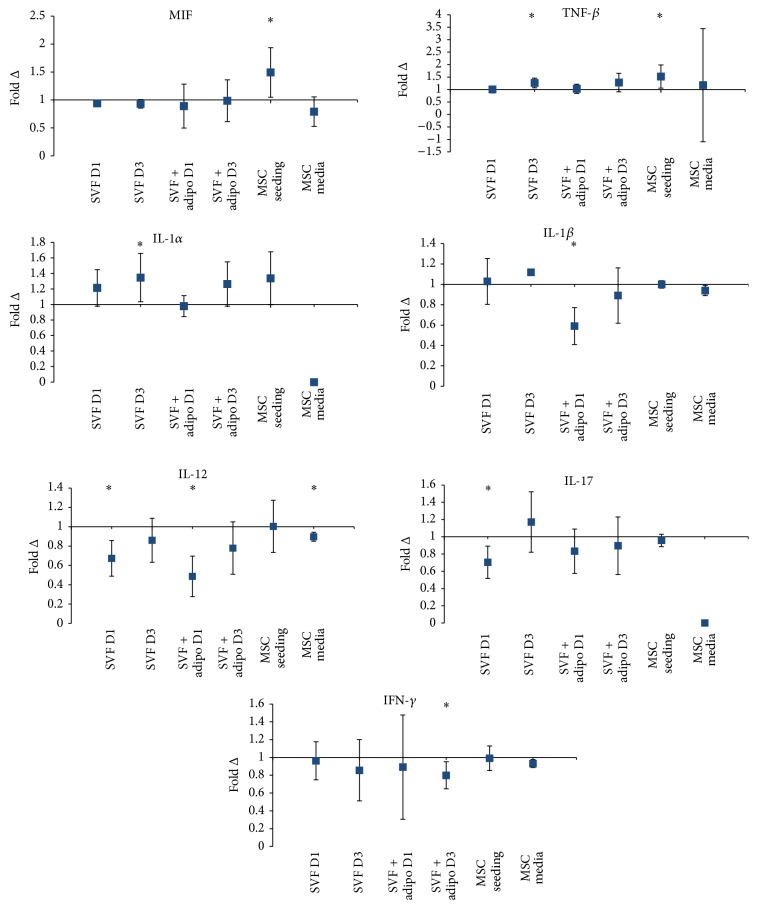
Fold change in concentration of proinflammatory cytokines detected across experiments Average fold change in concentration (*n* = 3) ± upper and lower confidence intervals at 95% (*y*-axis). Conditions (*x*-axis): stromal vascular fraction on day 1 (SVF D1), stromal vascular fraction on day 3 (SVF D3), stromal vascular fraction plus mature adipocytes on day 1 (SVF + adipo D1), stromal vascular fraction plus mature adipocytes on day 3 (SVF + adipo D3), seeding experiment with purified MSCs (MSC seeding), and media change experiment with purified MSCs (MSC media). Conditions were all cultured in either standard culture media or HA media. Cytokines with a fold change less than one indicate a decrease in concentration with HA media treatment; a fold change greater than one indicates an increase in the cytokine with HA treatment. Macrophage migration inhibitory factor (MIF), tumor necrosis factor-beta (TNF-*β*; lymphotoxin-alpha), interleukin-1*α* (IL-1*α*), interleukin-1*β* (IL-1*β*), interleukin-12 (IL-12), interleukin-17 (IL-17), and interferon-gamma (IFN-*γ*). IL-1*α* was not detected in the MSC media condition. IL-17 was only detected in one biological replicate in the MSC media condition. IL-1*β* was only detected in one biological replicate in the SVF condition on day 3.

The stromal vascular fraction (SVF) alone prepared from fresh lipoaspirate via enzymatic digestion is currently used in autologous therapy for the treatment of OA. When the SVF alone was combined with HA, proinflammatory cytokine IL-1*α* (Figure 4), increased 21% on the first day and significantly on the third day of culture by 34%. IL-12 decreased significantly on the first day by 33% (Figure 4) but later recovered on the third day of culture with a 15% decrease. IL-17 decreased significantly by 30% (Figure 4) when combined with HA on the first day of culture but on the third day increased by 17%.

FGF-*β* is one of six detected growth factors across the range of conditions tested. On the third day of culture, the SVF increased FGF-*β* secretion by 20% on the third day (Figure 6). Also on the third day of culture the SVF decreased secretion of GM-CSF significantly by 81% (Figure 6). VEGF also decreased on the first and third day culture by 21% and 19%, respectively, but not significantly (Figure 6).

Chemokines are chemotactic cytokines responsible for homing and immune cell recruitment. RANTES in the first day of culture in the SVF decreased by 23% but not significantly (Figure 7). The SVF on day one decreased secretion of MCP-1 by 21% and then significantly on the third day by 36% (Figure 7). MIP-1*α* decreased by 17% on the first day of SVF culture and then significantly on the third day of culture by 33% (Figure 7).

### 3.4. Stromal Vascular Fraction Plus Adipocytes

Aside from the stromal vascular fraction (SVF), another by-product of the enzymatic digestion of adipose tissue is mature adipocytes. A combination of the SVF and adipocytes can be used to treat knee OA. This therapy is available commercially for use in the autologous setting and has been used in a placebo control trial. In this study, SVF plus adipocytes were combined with HA and in the first day IL-*β* decreased significantly by 41% but then later recovered on the third day to just a 11% decrease (Figure 4). Also another very significant decrease was observed on the first day of culture, and the SVF plus mature adipocytes decreased the secretion of IL-12 by 52% when combined with HA, and this later recovered on the third day to just a 22% decrease (Figure 4).

**Figure 5:**
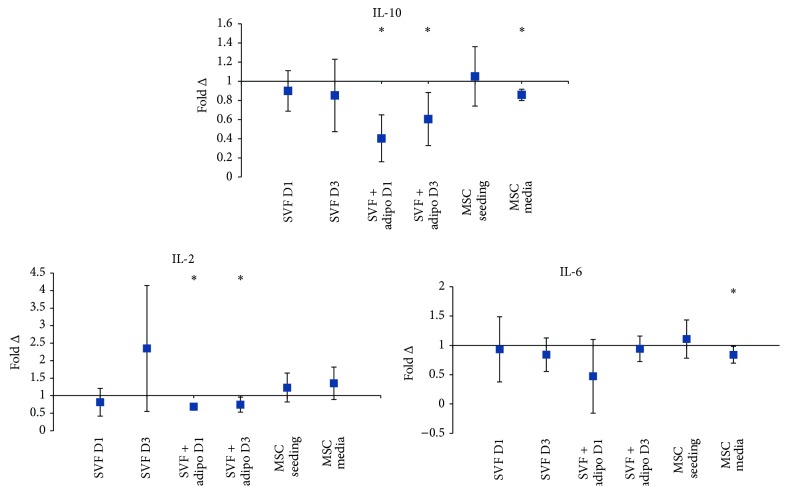
Fold change in concentration of anti-inflammatory and dual role cytokines detected across experiments. Average fold change in concentration (*n* = 3) ± upper and lower confidence intervals at 95% (*y*-axis). Conditions (*x*-axis): stromal vascular fraction on day 1 (SVF D1), stromal vascular fraction on day 3 (SVF D3), stromal vascular fraction plus mature adipocytes on day 1 (SVF + adipo D1), stromal vascular fraction plus mature adipocytes on day 3 (SVF + adipo D3), seeding experiment with purified MSCs (MSC seeding), and media change experiment with purified MSCs (MSC media). Conditions were all cultured in either standard culture media or HA media. Cytokines with a fold change less than one indicate a decrease in concentration with HA media treatment, and a fold change greater than one indicates an increase in the cytokine with HA treatment. Interleukin-10 (IL-10), interleukin-2 (IL-2), and interleukin-6 (IL-6).

Anti-inflammatory cytokine IL-10 decreased significantly on the first (60%) and on the third day (40%) of the SVF plus adipocytes combined with HA culture (Figure 5). The SVF plus adipocytes combined with HA also decreased secretion of IL-2 significantly on the first day by 31.5% and again on the third day significantly by 26% (Figure 5).

**Figure 6:**
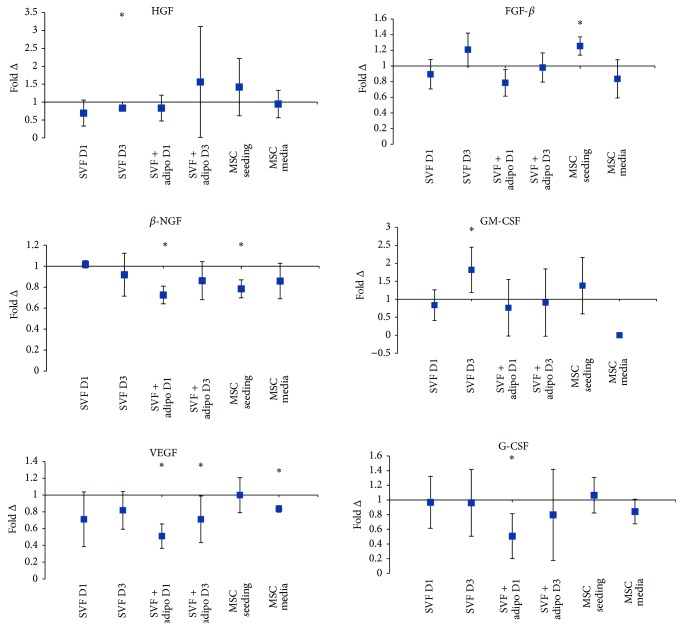
Fold change in concentration of growth factors detected across the experiments. Average fold change in concentration (*n* = 3) ± upper and lower confidence intervals at 95% (*y*-axis). Conditions (*x*-axis): stromal vascular fraction on day 1 (SVF D1), stromal vascular fraction on day 3 (SVF D3), stromal vascular fraction plus mature adipocytes on day 1 (SVF + adipo D1), stromal vascular fraction plus mature adipocytes on day 3 (SVF + adipo D3), seeding experiment with purified MSCs (MSC seeding), and media change experiment with purified MSCs (MSC media). Conditions were all cultured in either standard culture media or HA media. Cytokines with a fold change less than one indicate a decrease in concentration with HA media treatment, and a fold change greater than one indicates an increase in the cytokine with HA treatment. Hepatocyte growth factor (HGF), fibroblast growth factor-basic (FGF-*β*), nerve growth factor-beta (*β*-NGF), granulocyte macrophage colony-stimulating factor (GM-CSF), vascular endothelial growth factor (VEGF), and granulocyte colony-stimulating factor (G-CSF). GM-CSF was not detected across all biological replicates in the MSC media condition.

Growth factor secretions were also measured from the SVF plus adipocytes when combined with HA. *β*-FGF on the first day of culture decreased significantly by 21% but later recovered by the third day culture to no change from the control (Figure 6). *β*-NGF decreased significantly on the first day of culture by 27.5% but on the third day of culture was not significant and only decreased by 14% (Figure 6). VEGF secretion by the SVF plus adipocyte preparation when combined with HA decreased significantly by 50% and 29% on the first and third day of coculture, respectively (Figure 6). G-CSF also decreased significantly on the first day by 50% but then later recovered on the third day to decrease secretion not significantly by 20% (Figure 6).

**Figure 7:**
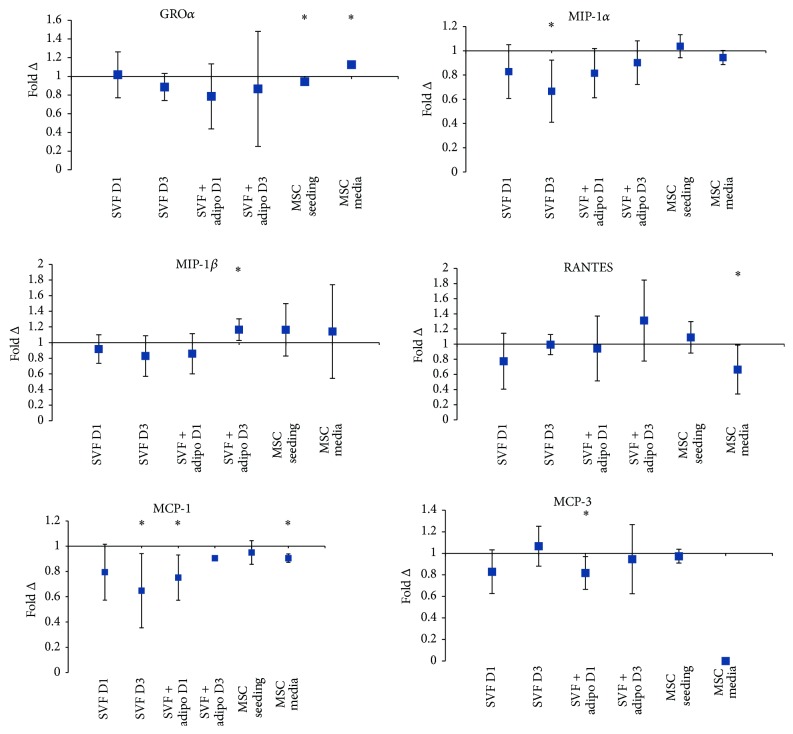
Fold change in concentration of chemokines detected across the experiments. Average fold change in concentration (*n* = 3) ± upper and lower confidence intervals at 95% (*y*-axis). Conditions (*x*-axis): stromal vascular fraction on day 1 (SVF D1), stromal vascular fraction on day 3 (SVF D3), stromal vascular fraction plus mature adipocytes on day 1 (SVF + adipo D1), stromal vascular fraction plus mature adipocytes on day 3 (SVF + adipo D3), seeding experiment with purified MSCs (MSC seeding), and media change experiment with purified MSCs (MSC media). Conditions were all cultured in either standard culture media or HA media. Cytokines with a fold change less than one indicate a decrease in concentration with HA media treatment, and a fold change greater than one indicates an increase in the cytokine with HA treatment. Chemokine ligand-1 (CXCL-1) aka growth-regulated peptide alpha (GRO*α*), CC-Chemokine ligand-4 (CCL-4) aka macrophage inflammatory protein-1 beta (MIP-1*β*), CC-Chemokine ligand-5 (CCL-5) aka regulated on activation normal T cell expressed and secreted (RANTES), CC-chemokine ligand-2 (CCL-2) aka monocyte chemotactic protein-1 (MCP-1), CC-chemokine ligand-7 (CCL-7) aka monocyte chemotactic protein-3 (MCP-3), and CC-chemokine ligand-3 (CCL-3) aka macrophage inflammatory protein-1-alpha (MIP-1*α*). MCP-3 was not detected across all biological replicates in the MSC media condition. MCP-1 was only detected in one of three biological replicates in the SVF + adipo D3 condition.

MIP-1*β* is one of six detectable chemokines across all the preparations. Secretion of MIP-1*β* by the SVF plus adipocytes when combined with HA decreased on the first day but on the third day increased significantly by 16.5% (Figure 7). MCP-1 decreased significantly on the first day when combined with HA by 25% on the third day and recovered to just a 10% decrease, and it was however only detected in one of three biological replicates (Figure 7). Finally MCP-3 decreased significantly by 18% on the first day when combined with HA but on the third day recovered with no observable change (Figure 7).

### 3.5. Culture Purified MSCs

The final preparation tested in this study and a likely future therapeutic in MSC therapy is the culture purified and expanded MSC population. In the MSC seeding experiment the combination with HA saw a significant increase in a major proinflammatory cytokine MIF by 49% but then a nonsignificant decrease after 3 days in the MSC media experiment of 21% (Figure 4). TNF-*β* secretion by purified MSCs in the MSC seeding experiment increased significantly by 52% when combined with HA but on the third day recovered to a 17% increase (Figure 4). IL-1*α* increased in the MSC seeding experiment by 34% but not significantly (Figure 4). There was no detectable IL-1*α* in the MSC media experiment.

IL-6 is considered a dual role cytokine and in the MSC seeding experiment increased by 10.5% but then decreased significantly in the MSC media experiment by 16% (Figure 5).

Culture purified MSCs in combination with HA also secrete appreciable amounts of growth factors. FGF-*β* increased significantly in the MSC seeding experiment by 25.5% but decreased in the MSC media by 16.5% (Figure 6). In the MSC seeding experiment *β*-NGF decreased significantly by 21.5% but in the MSC media experiment decreased nonsignificantly by 14% (Figure 6).

Chemokine secretion by culture purified MSCs in combination with HA was also measured. GRO*α* decreased significantly in the MSC seeding experiment by 6% but inversely significantly increased in the MSC media experiment by 12% (Figure 7). RANTES secretion did not change in the MSC seeding experiment; however in the MSC media experiment it decreased by 33.5% when combined with HA (Figure 7). MCP-1 showed no appreciable change in secretion in the MSC seeding experiment but decreased significantly by 9.5% in the MSC media experiment when combined with HA (Figure 7).

## 4. Discussion

Biological therapeutics such as MSC therapy are gaining acceptance for the treatment of inflammatory conditions, including musculoskeletal ailments such as OA.

It is likely that MSC therapy will be administered with viscosupplementation such as HA, which has extensive evidence for therapeutic efficacy in OA. In this study we used a number of adipose MSC preparations that are currently in commercial use or undergoing clinical trial for the treatment of OA. We cocultured the different cell preparations with HA to investigate the effects on the secretome. We found that MSCs maintained differentiation potential when cocultured with HA. These results are consistent with [34] although enhanced adipogenic potential was observed in the HA treated MSCs (data not shown). Immunophenotypic expression was characteristic of multipotent mesenchymal stem cells following coculture, and additionally, no observable changes could be observed in the expression of the hyaluronan receptor CD44.

Changes in the cytokine profile may be an indication of therapeutic efficacy reflective of combining HA and MSCs. We therefore profiled proinflammatory cytokines in an attempt to measure the effects of HA on MSCs either in stromal vascular fraction (SVF), SVF plus mature adipocytes, and culture MSC populations. Significant reductions in a well-established proinflammatory cytokine and interferon gamma in the SVF plus adipocyte preparation (Figure 4) suggest that this is a favorable preparation, although the fold change of all other preparations trended downward also. Interleukin-12 also trended downward for all preparations and significantly for the SVF, SVF plus adipocytes, and the MSC media. Interleukin-12 is expressed in the OA milieu [41] by infiltrating immune cells such as mature dendritic cells [42]; however MSCs are known to inhibit the maturation of dendritic cells [43] and thus a combination with HA for this cytokine may be desirable. The MSC seeding preparation was intended to model co-intra-articular injection of purified MSCs with HA. The significant increase in macrophage migration inhibitory factor (MIF) (Figure 4) may contraindicate the combination of purified MSCs with HA. Animal models have shown a correlation with reduced severity of OA and depletion of MIF [44]. MIF acts in local tissue to increase neutrophil and macrophage migration to regions of inflammation. Recent investigations have shown that TNF-*β* (lymphotoxin-alpha) initiates an inflammatory response in human chondrocytes as a consequence of increase NF-*κ*B signaling [45]. Figure 4 shows that there is both a significant increase of TNF-*β* in both the SVF alone and MSC seeding preparations, although the SVF plus adipocytes increased but not significantly, further supporting the SVF plus adipocytes as an ideal preparation. The secretion of interleukin-1 alpha (IL-1*α*) trended upward for most preparations, however only significantly for the SVF alone preparation. Interleukin-1 family of cytokines have long been implicated in degradation of the knee joint cartilage in OA [9], pending* in vivo* trials, and this increase may amplify endogenous interleukin-1 secretion in the OA joint much to the detriment of MSC therapy. Interleukin-1 beta (IL-1*β*) is heavily implicated in cartilage catabolism [46]. Collagen-induced arthritis models in mice have shown that HA alone can reduce IL-1*β* mRNA expression in articular cartilage [47]. Here we have shown minimal changes in purified MSC secretion of IL-1*β* when combined with HA; however when the SVF plus mature adipocytes were exposed to HA there was a significant 41% (Figure 4) decrease in secretion of IL-1b as opposed to an increase in secretion when the SVF alone was combined with HA. This may contraindicate the use of the SVF alone in combination with HA but encourage the addition of mature adipocytes.

Anti-inflammatory cytokines and growth factors play a crucial role in the regenerative potential of MSCs. As such, a decrease in anti-inflammatory cytokines as a result of the combination with HA may not be desirable in the context of trophic efficacy. Interleukin-10 decreased significantly in both SVF plus adipocytes and cultured MSC media preparation (Figure 5). Interleukin-10 secretion by MSCs has been proposed to be a cytokine involved in the inhibition of immune cell, Th17, and differentiation [48]. Therefore a downward trend in the preparation when combined with HA, as seen in Figure 5, may not be desirable for MSC therapy as it may hinder the immunomodulatory capacity. Basic fibroblast growth factor (FGF-*β*) increased significantly in the cultured MSC seeding preparation (Figure 6) which could suggest that increased proliferative capacity of MSCs as FGF-*β* is a potent mitogen. It may also play a synergistic role in regenerating cartilage in OA with MSCs. Animal models of cartilage defects have shown recombinant human FGF-*β* alone potentiates articular cartilage resurfacing after just 6 weeks [21]. However other studies suggest that endogenous FGF-*β* stimulates the expression of matrix metalloproteinase-13 [22], an enzyme which degrades type II collagen present in articular cartilage. Granulocyte macrophage colony-stimulating factor (GM-CSF) significantly increased in the SVF alone preparation (Figure 6). This change may negatively affect MSC therapy for the treatment of OA as GM-CSF is a key mediator in inflammation and arthritic pain [24]. Animal studies of experimental osteoarthritis using knockout mice showed that the development of OA was strictly dependent on GM-CSF [49]. Nerve growth factor-beta (*β*-NGF) showed a downward trend in most preparations and significantly in the SVF plus adipocytes and the cultured MSC seeding preparations (Figure 6). *β*-NGF in human osteoarthritic-derived chondrocytes shows increased mRNA expression when compared to chondrocytes from healthy donors [23] suggesting that the combination of HA with MSCs might be beneficial with decreased overall levels of *β*-NGF. Vascular endothelial growth factor (VEGF) is expressed in osteoarthritic-derived chondrocytes and shown to increase osteochondral angiogenesis in OA patients [25]. Animal models have shown that intra-articular injection of recombinant VEGF causes cartilage degradation as in OA [50]. VEGF showed a downward trend across all preparations and decreased significantly in the SVF plus adipocyte and cultured MSC media preparations when combined with HA (Figure 6). Colony stimulating factors are pivotal in influencing cartilage metabolism; a combination of IL-1*β* and granulocyte colony stimulating factor (G-CSF) significantly increased nitrite levels by cartilage explants [26]. In the SVF plus mature adipocytes preparation G-CSF secretion decreased 50% when combined with HA, the other preparation showed no appreciable changes. This again reiterates the SVF plus mature adipocyte preparation as an ideal combination with HA.

Chemokines are chemoattractant cytokines that play a pivotal role in regulating migration and infiltration of immune cell populations. In a study of OA-derived chondrocytes it was found that monocyte chemotactic protein-1 (MCP-1) induced matrix metalloproteinase-3 expression and inhibited proteoglycan synthesis [19]. MCP-1 in OA-derived subchondral bone marrow stromal cells also showed constitutive expression [51]. MCP-1 secretion decreased across all preparations and significantly for the SVF alone and SVF plus mature adipocytes by 35% and 25%, respectively. This suggests that a combination of these preparations with HA may synergistically work to reduce immune cell recruitment. Macrophage inflammatory protein-1-alpha (MIP-1*α*) showed increased expression in OA-derived bone marrow stromal cells when challenged with IL-1*β* [51]. Therefore modulating levels of MIP-1*α* is desirable for the treatment of OA. Secretion of this chemokine decreased across both the SVF alone and SVF plus adipocyte preparation but not appreciably in the purified MSC preparations.

## 5. Conclusion

In this study we investigated a range of adipose-derived mesenchymal stem cell preparations, some of which are currently used for autologous therapy and some of which will become important for future off-the-shelf allogeneic preparations. As the mode of action of MSCs is driven by the secretion of immunomodulatory and trophic factors, we assessed changes in the secretion of cytokines in the conditioned media when cells were cocultured with HA. When cocultured with HA, SVF plus mature adipocytes showed the greatest decrease in the proinflammatory cytokines IL-1*β*, IFN-*γ*, and the growth factor VEGF, which has been identified as a negative influence in OA. Two chemokines, MCP-1, and MIP-1*α* decreased substantially in the HA SVF preparations, with and without adipocytes, but not the purified MSCs. Both the SVF alone and purified MSC populations also had small but significant increases in TNF-*β* when cocultured with HA. There was an increase in TNF-*β* in the HA coculture of SVF and adipocytes, but this was not significant.

The purified MSCs cocultured with HA were the only preparation to show increased MIF, a major proinflammatory cytokine, which is correlated with OA severity. The increased concentration was observed at the initial stage of cell seeding and MIF was below the control concentration by the media change at day three. It is unknown what effect a transient spike of MIF may have on OA pathology and this would be ideally tested in an animal model.

As shown in previous* in vitro* and* in vivo* studies from our group, the mixed population of cells in adipose SVF plus adipocytes produces a distinct and therapeutically superior cytokine profile. In this study the SVF plus mature adipocytes preparation appears to be most suited of all the preparations for combination with HA.

## Figures and Tables

**Figure 1:**
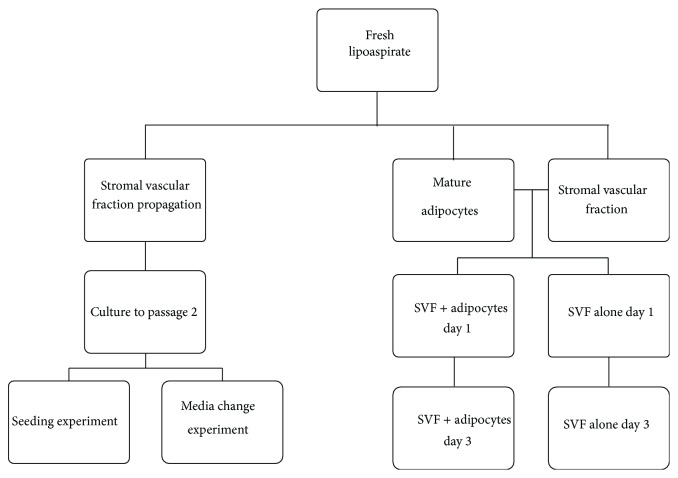


**Table 1:** Summary of cytokines across all conditions.

Cytokines	Fold change from control (CI = 95%)						Role in OA
	SVF day 1	SVF day 3	SVF + adipo day 1	SVF + adipo day 3	MSC seeding	MSC media	
Proinflammatory							
MIF	0.94	0.93	0.89	0.99	**1.49**	0.79	Induce the release of proinflammatory cytokines, such as TNF-*α*, IFN-*γ*, IL-1*β*, IL-6, and IL-8 [6] and MIF levels in synovial fluid correlate to OA severity [7]
TNF-*β*	1.00	**1.26**	1.03	1.28	**1.52**	1.18	TNF-beta stimulates cartilage matrix breakdown [8]
IL-1*α*	1.21	**1.35**	0.98	1.26	1.34	N.D.	Highly expressed in OA-derived human articular cartilage [9]
IL-1*β*	1.03	*1.12 *	**0.59**	0.89	1.00	0.94	Induce inflammatory reactions and catabolic effects independently as well as in combination with other mediators in OA with respect to the articular cartilage [10]
IL-12	**0.67**	0.86	**0.49**	0.78	1.00	**0.90**	Expressed by infiltrating macrophages and synovial cells in OA [11]
IL-17	**0.70**	1.17	0.83	0.90	0.96	*0.09 *	Inhibit the synthesis of proteoglycans in OA and upregulate catabolic enzymes which break down cartilage [10]
IFN-*γ*	0.96	0.86	0.89	**0.80**	0.99	0.93	Stimulate cartilage breakdown by production of enzymes via IL-1*β* [12]

Anti-inflammatory/dual role							
IL-10	0.90	0.85	**0.40**	**0.61**	1.05	**0.86**	Expressed in chondrocytes and involved in collagen and aggrecan synthesis. Inhibition of catabolic enzymes and apoptosis of chondrocytes [10]
IL-2	0.81	2.35	**0.69**	**0.74**	1.23	1.35	Elevated levels are found in OA synovial fluid and increased levels correlate to increase severity [13]
IL-6	0.93	0.84	0.47	0.94	1.11	**0.84**	Catabolism aggrecan via aggrecanase activity [14] involved in the synthesis of tissue inhibitor of metalloproteinases known to stop cartilage breakdown [15]

Chemokines							
GRO*α*	1.02	0.89	0.79	0.87	**0.94**	**1.12**	Constitutively secreted by chondrocytes and secretion of this chemotactic protein is increased in stimulated osteoarthritic chondrocytes and synovial fibroblasts [16]
MIP-1*β*	0.92	0.83	0.86	**1.17**	1.16	1.14	Present in significantly higher levels in OA synovial fluid compared to normal synovial fluid [17]
RANTES	0.78	0.99	0.94	1.31	1.09	**0.67**	Secreted by IL-1 stimulated osteoarthritic chondrocytes and TNF-*α* stimulated synovial fibroblasts [16]
MCP-1	0.79	**0.65**	**0.75**	*0.90 *	0.95	**0.91**	Constitutively expressed in osteoarthritic chondrocytes and increased secretion in IL-1 stimulated osteoarthritic chondrocytes [16]
MCP-3	0.83	1.07	**0.82**	0.95	0.97	N.D.	Activate immune cells such as monocytes, T lymphocytes, basophils, and eosinophils [18]
MIP-1*α*	0.83	**0.67**	0.82	0.90	1.04	0.95	Expressed by osteoarthritic derived chondrocytes and production is enhanced by TNF-*α* stimulated chondrocytes [19]

Growth factors							
HGF	0.69	**0.83**	0.83	1.56	1.42	0.95	Promote osteophyte formation via MCP-1 mediated infiltration of immune cells into OA affected joint [20]
FGF-*β*	0.89	1.21	**0.79**	0.98	**1.26**	0.84	Potentiate articular cartilage resurfacing and may stimulate expression of MMP-13 [21, 22]
*β*-NGF	1.02	0.92	**0.73**	0.86	**0.78**	0.86	Higher expression is found in osteoarthritic derived chondrocyte compared to healthy chondrocytes [23]
GM-CSF	0.84	**1.82**	0.76	0.91	1.38	N.D.	Key mediator in inflammation and arthritic pain [24]
VEGF	0.71	0.82	**0.51**	**0.71**	1.00	**0.84**	Expressed in osteoarthritic-derived chondrocytes and shown to increase osteochondral angiogenesis in OA patients [25]
G-CSF	0.97	0.96	**0.51**	0.80	1.06	0.84	Can significantly increase nitrite levels in cartilage when combined with IL-1*β* explants [26]

A summary of cytokines across all the conditions treated with HA. The data is represented as fold change in concentration compared to control. A value more than one indicates the HA treatment increased the secretion of that cytokine in that condition. Bold values represent a significant fold change from control determined numerically using confidence interval set at 95%. Italic values were only detected in one of the three biological replicates for that condition.

## References

[1] Fraser J. R. E., Laurent T. C., Laurent U. B. G. (1997). Hyaluronan: its nature, distribution, functions and turnover. *Journal of Internal Medicine*.

[2] Balazs E. A., Watson D., Duff I. F., Roseman S. (1967). Hyaluronic acid in synovial fluid. I. Molecular parameters of hyaluronic acid in normal and arthritic human fluids. *Arthritis and Rheumatism*.

[3] Pauloin T., Dutot M., Warnet J.-M., Rat P. (2008). In vitro modulation of preservative toxicity: high molecular weight hyaluronan decreases apoptosis and oxidative stress induced by benzalkonium chloride. *European Journal of Pharmaceutical Sciences*.

[4] Le D. H., Goodman S. B., Maloney W. J., Huddleston J. I. (2014). Current modes of failure in TKA: infection, instability, and stiffness predominate. *Clinical Orthopaedics and Related Research*.

[5] Li C. S., Karlsson J., Winemaker M., Sancheti P., Bhandari M. (2014). Orthopedic surgeons feel that there is a treatment gap in management of early OA: international survey. *Knee Surgery, Sports Traumatology, Arthroscopy*.

[6] Leech M., Metz C., Hall P. (1999). Macrophage migration inhibitory factor in rheumatoid arthritis: evidence of proinflammatory function and regulation by glucocorticoids. *Arthritis & Rheumatism*.

[7] Liu M., Hu C. (2012). Association of MIF in serum and synovial fluid with severity of knee osteoarthritis. *Clinical Biochemistry*.

[8] Campbell I. K., Piccoli D. S., Roberts M. J., Muirden K. D., Hamilton J. A. (1990). Effects of tumor necrosis factor alpha and beta on resorption of human articular cartilage and production of plasminogen activator by human articular chondrocytes. *Arthritis and Rheumatism*.

[9] Towle C. A., Bonassar L. J., Treadwell B. V., Mangham D. C. (1997). Detection of interleukin-1 in the cartilage of patients with osteoarthritis: a possible autocrine/paracrine role in pathogenesis. *Osteoarthritis and Cartilage*.

[10] Wojdasiewicz P., Poniatowski A. A., Szukiewicz D. (2014). The role of inflammatory and anti-inflammatory cytokines in the pathogenesis of osteoarthritis. *Mediators of Inflammation*.

[11] Sakkas L. I., Johanson N. A., Scanzello C. R., Platsoucas C. D. (1998). Interleukin-12 is expressed by infiltrating macrophages and synovial lining cells in rheumatoid arthritis and osteoarthritis. *Cellular Immunology*.

[12] Rutgers M., Saris D. B. F., Dhert W. J. A., Creemers L. B. (2010). Cytokine profile of autologous conditioned serum for treatment of osteoarthritis, *in vitro* effects on cartilage metabolism and intra-articular levels after injection. *Arthritis Research & Therapy*.

[13] Vangsness C. T., Burke W. S., Narvy S. J., MacPhee R. D., Fedenko A. N. (2011). Human knee synovial fluid cytokines correlated with grade of knee osteoarthritis: a pilot study. *Bulletin of the NYU Hospital for Joint Diseases*.

[14] Flannery C. R., Little C. B., Hughes C. E., Curtis C. L., Caterson B., Jones S. A. (2000). IL-6 and its soluble receptor augment aggrecanase-mediated proteoglycan catabolism in articular cartilage. *Matrix Biology*.

[15] Lotz M., Guerne P.-A. (1991). Interleukin-6 induces the synthesis of tissue inhibitor of metalloproteinases-1/erythroid potentiating activity (TIMP-1/EPA). *The Journal of Biological Chemistry*.

[16] Honorati M. C., Bovara M., Cattini L., Piacentini A., Facchini A. (2002). Contribution of interleukin 17 to human cartilage degradation and synovial inflammation in osteoarthritis. *Osteoarthritis and Cartilage*.

[17] Koch A. E., Kunkel S. L., Shah M. R. (1995). Macrophage inflammatory protein-1*β*: a C-C chemokine in osteoarthritis. *Clinical Immunology and Immunopathology*.

[18] Xu L. L., Mc Vicar D. W., Ben-Baruch A. (1995). Monocyte chemotactic protein-3 (MCP3) interacts with multiple leukocyte receptors: Binding and signaling of MCP3 through shared as well as unique receptors on monocytes and neutrophils. *European Journal of Immunology*.

[19] Yuan G.-H., Masuko-Hongo K., Sakata M. (2001). The role of C–C chemokines and their receptors in osteoarthritis. *Arthritis & Rheumatism*.

[20] Dankbar B., Neugebauer K., Wunrau C. (2007). Hepatocyte growth factor induction of macrophage chemoattractant protein-1 and osteophyte-inducing factors in osteoarthritis. *Journal of Orthopaedic Research*.

[21] Fujimoto E., Ochi M., Kato Y., Mochizuki Y., Sumen Y., Ikuta Y. (1999). Beneficial effect of basic fibroblast growth factor on the repair of full-thickness defects in rabbit articular cartilage. *Archives of Orthopaedic and Trauma Surgery*.

[22] Wang X., Manner P. A., Horner A., Shum L., Tuan R. S., Nuckolls G. H. (2004). Regulation of MMP-13 expression by RUNX2 and FGF2 in osteoarthritic cartilage. *Osteoarthritis and Cartilage*.

[23] Iannone F., de Bari C., Dell'Accio F. (2002). Increased expression of nerve growth factor (NGF) and high affinity NGF receptor (p140 TrkA) in human osteoarthritic chondrocytes. *Rheumatology*.

[24] Cook A. D., Pobjoy J., Sarros S. (2013). Granulocyte-macrophage colony-stimulating factor is a key mediator in inflammatory and arthritic pain. *Annals of the Rheumatic Diseases*.

[25] Walsh D. A., Mcwilliams D. F., Turley M. J. (2010). Angiogenesis and nerve growth factor at the osteochondral junction in rheumatoid arthritis and osteoarthritis. *Rheumatology*.

[26] Stephan S., Purcell W. M., Chander C. L. (1999). Colony stimulating factors regulate nitric oxide and prostaglandin E2 production in rat cartilage chondrocytes. *International Journal of Tissue Reactions*.

[27] Clouet J., Vinatier C., Merceron C. (2009). From osteoarthritis treatments to future regenerative therapies for cartilage. *Drug Discovery Today*.

[28] Zuk P. A., Zhu M., Mizuno H. (2001). Multilineage cells from human adipose tissue: Implications for cell-based therapies. *Tissue Engineering*.

[29] Caplan A. I., Dennis J. E. (2006). Mesenchymal stem cells as trophic mediators. *Journal of Cellular Biochemistry*.

[30] Blaber S. P., Webster R. A., Breen E. J., Vesey G., Herbert B. R. (2013). Treatment of a mouse model of collagen antibody-induced arthritis with human adipose-derived secretions. *Open Journal of Regenerative Medicine*.

[31] Hedbom E., Häuselmann H. J. (2002). Molecular aspects of pathogenesis in osteoarthritis: the role of inflammation. *Cellular and Molecular Life Sciences*.

[32] Pelletier J.-P., Martel-Pelletier J., Abramson S. B. (2001). Osteoarthritis, an inflammatory disease: potential implication for the selection of new therapeutic targets. *Arthritis & Rheumatism*.

[33] Wu L., Prins H.-J., Helder M. N., Van Blitterswijk C. A., Karperien M. (2012). Trophic effects of mesenchymal stem cells in chondrocyte co-cultures are independent of culture conditions and cell sources. *Tissue Engineering—Part A*.

[34] Lee K. B. L., Hui J. H. P., Im C. S., Ardany L., Eng H. L. (2007). Injectable mesenchymal stem cell therapy for large cartilage defects—a porcine model. *Stem Cells*.

[35] Kern S., Eichler H., Stoeve J., Klüter H., Bieback K. (2006). Comparative analysis of mesenchymal stem cells from bone marrow, umbilical cord blood, or adipose tissue. *Stem Cells*.

[36] Ruppert S. M., Hawn T. R., Arrigoni A., Wight T. N., Bollyky P. L. (2014). Tissue integrity signals communicated by high-molecular weight hyaluronan and the resolution of inflammation. *Immunologic Research*.

[37] Blaber S. P., Webster R. A., Hill C. J. (2012). Analysis of in vitro secretion profiles from adipose-derived cell populations. *Journal of Translational Medicine*.

[38] Blaber S. P., Hill C. J., Webster R. A. (2013). Effect of labeling with iron oxide particles or nanodiamonds on the functionality of adipose-derived mesenchymal stem cells. *PLoS ONE*.

[39] Lesley J., Hyman R., Kincade P. W. (1993). CD44 and its interaction with extracellular matrix. *Advances in Immunology*.

[40] Dominici M., Le Blanc K., Mueller I. (2006). Minimal criteria for defining multipotent mesenchymal stromal cells. The International Society for Cellular Therapy position statement. *Cytotherapy*.

[41] Morita Y., Yamamura M., Nishida K. (1998). Expression of interleukin-12 in synovial tissue from patients with rheumatoid arthritis. *Arthritis and Rheumatism*.

[42] Nauta A. J., Kruisselbrink A. B., Lurvink E., Willemze R., Fibbe W. E. (2006). Mesenchymal stem cells inhibit generation and function of both CD34+-derived and monocyte-derived dendritic cells. *The Journal of Immunology*.

[43] Spaggiari G. M., Abdelrazik H., Becchetti F., Moretta L. (2009). MSCs inhibit monocyte-derived DC maturation and function by selectively interfering with the generation of immature DCs: central role of MSC-derived prostaglandin E2. *Blood*.

[44] Greene M. A., Roland A., Pritzker L. (2014). Deletion of macrophage migration inhibitory factor reduces severity of osteoarthritis in mice. *Osteoarthritis and Cartilage*.

[45] Buhrmann C., Shayan P., Aggarwal B. B., Shakibaei M. (2013). Evidence that TNF-*β* (lymphotoxin *α*) can activate the inflammatory environment in human chondrocytes. *Arthritis Research and Therapy*.

[46] Daheshia M., Yao J. Q. (2008). The interleukin 1*β* pathway in the pathogenesis of osteoarthritis. *The Journal of Rheumatology*.

[47] Campo G. M., Avenoso A., Nastasi G. (2011). Hyaluronan reduces inflammation in experimental arthritis by modulating TLR-2 and TLR-4 cartilage expression. *Biochimica et Biophysica Acta—Molecular Basis of Disease*.

[48] Qu X., Liu X., Cheng K., Yang R., Zhao R. C. H. (2012). Mesenchymal stem cells inhibit Th17 cell differentiation by IL-10 secretion. *Experimental Hematology*.

[49] Cook A. D., Pobjoy J., Steidl S. (2012). Granulocyte-macrophage colony-stimulating factor is a key mediator in experimental osteoarthritis pain and disease development. *Arthritis Research and Therapy*.

[50] Ludin A., Sela J. J., Schroeder A., Samuni Y., Nitzan D. W., Amir G. (2013). Injection of vascular endothelial growth factor into knee joints induces osteoarthritis in mice. *Osteoarthritis and Cartilage*.

[51] Lisignoli G., Toneguzzi S., Pozzi C. (1999). Chemokine expression by subchondral bone marrow stromal cells isolated from osteoarthritis (OA) and rheumatoid arthritis (RA) patients. *Clinical & Experimental Immunology*.

